# Michigan State Board of Health

**Published:** 1888-08

**Authors:** 


### MICHIGAN STATE BOARD OF HEALTH.

### Regular Meeting, July 10, 1888.

The regular quarterly meeting of the Michigan State Board of Health was held in Lansing, July 10, 1888.

Besides the routine business, certain proposed bills for perfecting the public health laws of the State were discussed and referred to the Committee on Legislation. The objects of the bills are as follows:

1. A Bill to prevent the use and sale of infected milk and infected-milk products;
2. A Bill to declare the least proportion of milk solids and of fat in unadulterated milk;
3. A Bill to require the labeling of all gasoline, benzine, and naphtha sold at reretail; and
4. A Bill to prevent the introduction and spread of dangerous communicable diseases.

Relative to Bill No. 2, mentioned above, it may be said that a law in Michigan prohibits the sale of impure or adulterated milk, and provides for an inspection of milk in Detroit and other cities and incorporated villages in the State. This law provides penalties for its violation, but it does not fully explain what is meant by pure milk. In each prosecution it is necessary to go over the whole ground of proving what should be taken as a standard, and why. If an unscrupulous chemist appears for the defense, and gives it as his opinion that the standard is too high, the offending milkman may escape because of this conflict of testimony. If the law fixed a definite legal standard, such a result would not be possible. The thousands of analyses made and recorded in the different States of the Union supply a basis for fixing such a legal standard.

The object of Bill No. 4, is to punish any one who, knowingly affected with small-pox, scarlet fever, diphtheria, or any other dangerous communicable disease, shall willfully enter a public place or a public conveyance, or who shall willfully go into any other locality without a permit of the board of health of that locality, or who shall knowingly and willfully convey any child or irresponsible person affected with any dangerous communicable disease, or the corpse of a person dead from such disease, or who shall, in any way, subject another to danger of contracting one of these diseases. At present there is no statutory provision punishing those traveling from place to place on the cars or in other public conveyance, knowing themselves to be affected with a dangerous communicable disease, and cases have been brought to the notice of the State Board of Health which would be covered by this proposed bill.

A letter was presented from a citizen of Branch County complaining that incompetent practitioners were driven, by the stringent laws of Indiana and other States, into the southern counties of Michigan, and asking the State Board of Health to exert its influence to protect the State from charlatans and quacks. The subject was discussed by the board and referred to a committee with instructions to draft a proposed bill for presentation to the Legislature.

DIETARY OF STATE INSTITUTIONS.

The secretary stated that replies had been received from seven different State institutions in reply to the request made by the State Board of Health for information concerning dietary. The replies were read at a meeting of the board, and were referred to Dr. Hazlewood for further careful study and report.

POISONING BY TYROTOXICON.

A communication was read from Dr. W. C. West, of Monroe, concerning about twenty cases of cheese-poisoning in that city. Those poisoned "were taken at first with great prostration—a sensation as though they would die. This was followed by vomiting which would relieve them somewhat. The vomiting was very severe in most of the cases, and was followed by diarrhœa. There was pain in the stomach and bowels." All who ate of the cheese were affected.

A sample of the cheese was sent to Professor R. C. Kedzie for analysis, who found that poison present in the sample, and attributed its poisonous effect to tyrotoxicon.

Complaints were made to the board that some health-officers use their official position to injure brother practitioners who report dangerous communicable diseases, by going to a case after the prominent symptoms have disappeared, deciding that it is not the disease reported, and thus bringing upon the physician who reported it the contempt of the family. After considerable discussion, the following resolution was adopted:

*Resolved*, That it is the opinion of this board, when a case of dangerous communicable disease is reported by a reliable physician, the health-officer should act upon the diagnosis of such physician without question.

COMPENSATION OF HEALTH-OFFICERS.

A card with printed questions was sent to each of 1,055 health-officers of townships, 213 health-officers of villages, 48 health-officers of cities (in all, 1,316 localities) asking for information concerning the compensation of health-officers, work performed by them, etc. Replies were received from 551 townships, 104 villages, and 20 cities—in all 675 localities.

According to the reports, provision is made for an *annual salary* in 111 townships, 54 villages, and 18 cities. The annual salaries provided for average $16.93 for each township, $128.33 for each city, and $26.21 for each village.

HOW WELL DOES PUBLIC OPINION SUSTAIN PUBLIC-HEALTH WORK?

A large majority—444 of the 593 health-officers—stated that they were well sustained; 151 said that they were poorly sustained, and 36 stated that public opinion did not sustain them at all.

SERVICES RENDERED BY THE HEALTH-OFFICERS.

Replies to the question as to services rendered by the health-officer were received from 593 localities. The number of nuisances inspected in all the localities reported was 5,075; the number abated was 3,679. The number of reports made to the State Board of Health was 4,029, and the number of communicable diseases looked after was 5,389. The above numbers do not fully show the amount of service performed, because many health-officers stated that they had inspected and abated nuisances, sent reports to the State Board of Health, and looked after cases of communicable diseases, but had kept no record in either case; therefore the foregoing numerical statements of work performed are too small; and they represent only 593 out of the 1,494 localities in the State.

EXAMINATION OF PLANS FOR A PROPOSED STATE BUILDING.

The plans for a proposed cottage for convalescent insane near the asylum at

Kalamazoo were examined at considerable length, but not very satisfactory results could be reached because of lack of statement of details. Letters explanatory by the medical superintendent, Dr. Palmer, were read by the secretary, and these letters gave more information than did the plans themselves.

The proposed cottage is to accommodate fifty inmates. The plans and letters show that considerable attention has been given to some of the sanitary requirements.

QUARTERLY REPORT OF WORK.

During the second quarter of 1888, the office of the board received information of and took action concerning 27 outbreaks of typhoid fever, 59 outbreaks of diphtheria, and 84 outbreaks of scarlet fever. To the localities where the outbreaks occurred, the usual number of documents on the restriction and prevention of these diseases were sent for distribution to those most interested. The office also received information concerning 91 outbreaks of measles.

